# Hospitals without Medical Schools

**Published:** 1889-07-20

**Authors:** 


### Hospitals Without Medical Schools.

### UNCONSIDERED TRIFLES.

"The farther we go from London, the cheaper it seems to be to maintain a hospital." These are the words of "The Hospital Annual," and we want, like the twenty thousand Cornishmen, to "know the reason why." For this purpose it is better to take hospitals without medical schools attached to them. Where there is a medical school and a teaching staff there must, as we have pointed out, be a certain amount of extra expenditure. Some of this is necessary, some is inevitable, some, we venture to say, is neither. Of the latter kind is a case we heard of recently. A surgeon at a London hospital invented a new kind of splint, and, pleased with his invention, ordered a large number to be made after the same pattern—"enough to fill a cellar," our informant said. The new invention proved to be useless for all but a very few cases, and in the cellar the splints still remain. Such cases may not be numerous, but still all hospital doctors should feel the responsibility that lies upon them in spending the public money, and while sparing nothing that will conduce to the patients' well-being, should be careful not to indulge in uncalled-for expenditure.

The instance we have quoted is one that would occur only in a teaching hospital, but those who examine hospitals closely will often find carelessness in minor points of expenditure. Recently, we went over a hospital, situated in a poor and populous neighbourhood, which cannot be fully furnished for lack of funds. In an empty ward we saw a large heap of crutches, splints, etc., lying on the floor among empty boxes and other rubbish. "Oh," said the house surgeon, our companion, "I wish I had known of these. We have been keeping a man in hospital for nearly a week because we hadn't a pair of crutches to lend him." Here, in a poor hospital was a double loss—the crutches and the rest lying useless and rotting, while the institution had the needless cost of the patient's keep, who, but for the difficulty of providing him with some aid in walking, would have gone out, and, perhaps, left a bed open for some more pressing case. The instance is not an important one, it may be; but large extravagance is the least thing we are likely to meet with in hospital expenditure. Economy, and even parsimony, in large matters is quite compatible with extravagance in small ones, and may even be the cause of it. Nurses and wardmaids occasionally tell how a matron refuses to give them a sufficiency of dusters, the result being that pieces of lint are used for dusting, and then, lest it should come out that they have been employed for such a purpose, thrown into the fire. We are perhaps dilating on trifles, but it is because we are sure that in these trifles will be found the real cause of differences of cost in hospital management which are otherwise inexplicable.

Often it is difficult to know who to blame. Perhaps the honorary medical staff escape criticism more than others, simply because their skilled services are gratuitous. It is too much like looking a gift horse in the mouth to complain of Dr. Brown, who has wrought such splendid cures, because Dr. Jones's patients do not cost so much per diem for food, and Dr. Robinson's do not average such a long stay in hospital. Nor would anyone wish Dr. Brown to deprive his patients of comforts that they need; but it cannot be denied that some doctors, out of easy good nature, order for their patients indulgence which they do not require. In some Scottish hospitals there is an idea that the doctors do occasionally let good-nature outrun discretion, and it is customary to compare the diet sheets of Dr. A.'s ward with those of Dr. B.'s. The result has been a considerable increase in economy; and though it is possible that the reform may go too far, and a parsimonious rivalry be set up between the doctors, this is much less likely to occur than the easy-going generosity with the subscribers' money, to which the medical staff are more liable than any other hospital officials.

But to return to our text: "The farther we go from London, the cheaper it seems to be to maintain a hospital." Food is not dearer in London than elsewhere, ward furniture is on the whole cheaper. The only reason we know of that would account for the total expenditure being greater in the metropolis than in the country is that London hospitals have to pay rates, a thing which, we think, they might well be spared. For the rest, greater efficiency is the only excuse for greater expenditure, and on this point only personal knowledge will fully justify comparison; therefore we will speak with reserve. But a recent visit to Leicester Infirmary convinced us that it was well managed; that the patients were happy and comfortable; that the nursing was good, and that the nurses were well treated. We may take it as a favourable specimen of a provincial hospital. The annual cost there per occupied bed was £52 17s. 9d., or £3 1s. 1d. per in-patient. (This is the calculation given in the tables of comparison in "The Hospital Annual." The secretary's return gives £2 18s. per in-patient). The lowest cost per occupied bed in London is at the Seamen's, where it averages £59 4s. 4d. per annum, or £5 0s. 5d. for each in-patient.

We have not chosen Leicester as a specially economical hospital, but only as one in which, to our knowledge, comfort is not sacrificed to economy. Nothing can be less desirable than pinching in hospital maintenance, and we would be most unwilling to deify undue economy, therefore we choose as an example an institution which, to our knowledge, is managed on generous lines. The Royal Southern Hospital, Liverpool, has a smaller average cost per bed—namely, £44 15s. 4d., and the Swansea Hospital gives a return of only £33 5s. 9d. per occupied bed. At the other extreme stands the Royal Portsmouth, Portsea and Gosport Hospital, with a cost per occupied bed of £107 8s. 2d. We sincerely hope that the Portsmouth Hospital is in every respect three times as good as Swansea, and twice as good as Leicester. If not, we should suggest that the officials examine their various departments very carefully to be sure that such a large expenditure is justified.

The difference in provisions alone is very great. In Swansea the cost is £10 per head, in Hartlepool £23, at Stockport £30. Do the Swansea patients eat so much less than their fellows at Stockport, or it is a question of contract going on from year to year without investigation, while the prices of the articles supplied vary continually? In the latter case one thing is evidently necessary, a strict and constant scrutiny on the part of secretary, steward, and governors.

In this connection we may mention two points on which we hold strong convictions. First, that no member of the house committee of a hospital should supply goods of any sort to it; where that is permitted it is expecting too much of human nature to hope that no abuses will creep in. Second, that some of the medical staff of the hospital should, *ex officio*, be members of committee. We have already hinted that they may be too lavish in their orders for patients' comforts, but this very tendency will act as a corrective to any desire for undue economy, at the cost of efficiency, which might prevail among other members less cognisant of the needs of the sick.

The expenditure on surgery and dispensary varies greatly. At Darlington it is £12 19s. 11d. per occupied bed, at Sunderland only £2 10s. This may be partly explained by the fact that Darlington has a dispensary attached to the hospital. A great part of the sum we have quoted is expended on out-patients, and therefore should not, by rights, be stated as "per occupied bed." But until the elimination of the out-patient from the general accounts be accomplished, such misrepresentations must prevail. We must note, however, that in Scotland the expenditure on surgery and dispensary is twice as great as in Ireland. In Scotland the average is fairly equal and moderately high; the lowest being Paisley Infirmary with £3 2s. 10d. per bed, the highest the Northern Infirmary, Inverness, with £8 13s. 3d. In Ireland, the highest expenditure under this head is at Cork—£3 8s. 4d., while in the County Limerick Infirmary it is so little as 15s. 9d. We consider the latter suspiciously low. In Ireland, also, salaries are very small. To a certain extent, this may be accounted for by the general rate of wages in that country; but in many cases it means, we have reason to believe, an absence of a proper staff of trained nurses. Nothing is more discouraging to a nurse who knows and loves her work than to find that her fellow-workers and subordinates are narrow-minded and incompetent. In this matter it may be hoped that in very few hospitals is there much to complain of, and that before long no reasonable cause for dissatisfaction will remain.

There is a general tendency, too general, we think, to assume that provincial hospitals are inferior to metropolitan ones. We cannot say that all provincial institutions reach the high level of London ones, but in the larger towns, all over the country, we venture to say that a high ideal is sought and maintained. It is attained at a smaller cost than marks equal efficiency in London. The reasons for this we commend to the enquiring attention of our hospital officials, and shall be glad to hear their solution of the problem. But there is one reason which we think concerns the public. In no town does money cost so much to collect as in London. If subscribers would more frequently send the sums they mean to give without waiting for a reminder of some sort, they would effect a perceptible saving in hospital management, and would give now harassed officials time to investigate many matters and plan many economies.

